# Supplementary figures and images for: A new test suggests hundreds of amino acid polymorphisms in humans are subject to balancing selection
Source: PLoS Biol. 2022 Jun 2;20(6):e3001645. doi: 10.1371/journal.pbio.3001645 (PMC9162324; doi:10.1371/journal.pbio.3001645)

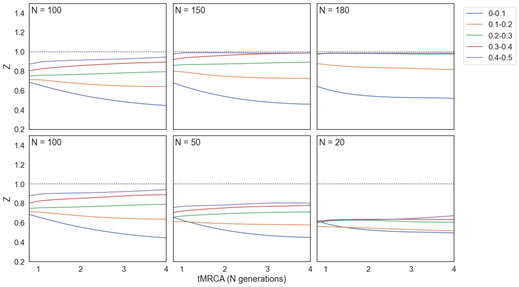

Supplement: S1 Fig — Each column is a separate set of simulations, with the top row plotting Z against tMRCA (measured in N generations, where N is the population size) for the larger daughter population, and the bottom row the smaller. There is no balancing selection and deleterious mutations are drawn from a gamma DFE with parameters inferred from human population data. Code to run these simulations can be found at https://github.com/vivaksoni/test_for_balancing_selection. DFE, distributions of fitness effect; tMRCA, time to the most recent common ancestor. (TIF) [file pbio.3001645.s004.tif]

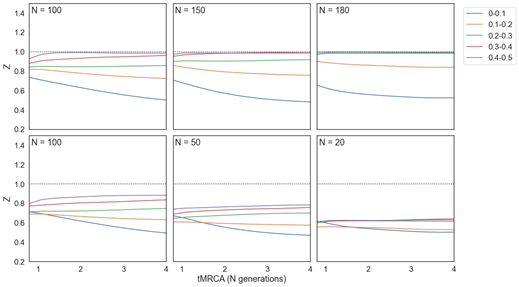

Supplement: S2 Fig — Each column is a separate set of simulations, with the top row plotting Z against tMRCA (measured in N generations, where N is the population size) for the ancestral population, and the bottom row the daughter population. There is no balancing selection and deleterious mutations are drawn from a gamma DFE with parameters inferred from human population data. Code to run these simulations can be found at https://github.com/vivaksoni/test_for_balancing_selection. DFE, distributions of fitness effect; tMRCA, time to the most recent common ancestor. (TIF) [file pbio.3001645.s005.tif]

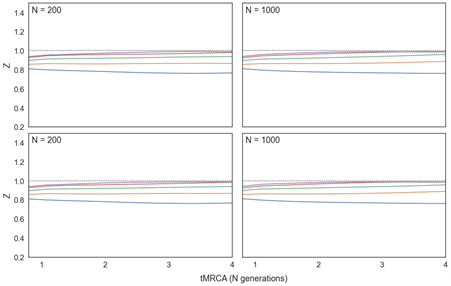

Supplement: S3 Fig — The ancestral population (of size N = 200) splits to form 2 daughter populations of size N = 100. Both daughter populations go on to expand in size. In the left column, the daughter populations double in size. In the right panel, they reach 10× their initial size. There is no balancing selection and deleterious mutations are drawn from a gamma DFE with parameters inferred from human population data. Code to run these simulations can be found at https://github.com/vivaksoni/test_for_balancing_selection. DFE, distributions of fitness effect; tMRCA, time to the most recent common ancestor. (TIF) [file pbio.3001645.s006.tif]

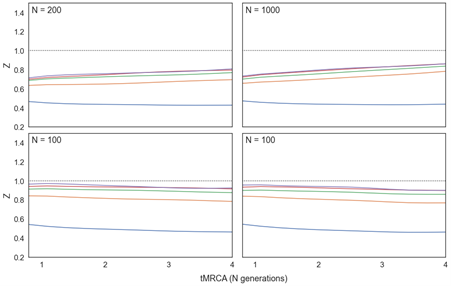

Supplement: S4 Fig — The ancestral population (of size N = 200) splits to form 2 daughter populations of size N = 100. One daughter population (upper panels) goes on to expand in size. In the left column, the daughter populations double in size. In the right panel, they reach 10× their initial size. There is no balancing selection and deleterious mutations are drawn from a gamma DFE with parameters inferred from human population data. Code to run these simulations can be found at https://github.com/vivaksoni/test_for_balancing_selection. DFE, distributions of fitness effect; tMRCA, time to the most recent common ancestor. (TIF) [file pbio.3001645.s007.tif]

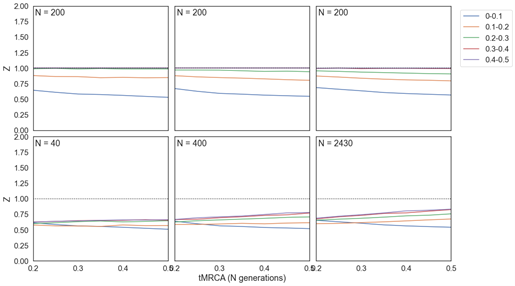

Supplement: S5 Fig — The ancestral population (of size N = 200) splits to form a daughter population of size N = 100, which expands to the final population size shown in the panel. Each column is a separate set of simulations, with the top row plotting Z against tMRCA (measured in N generations, where N is the population size) for the ancestral population, and the bottom row the daughter population. There is no balancing selection and deleterious mutations are drawn from a gamma DFE with parameters inferred from human population data. Code to run these simulations can be found at https://github.com/vivaksoni/test_for_balancing_selection. DFE, distributions of fitness effect; tMRCA, time to the most recent common ancestor. (TIF) [file pbio.3001645.s008.tif]

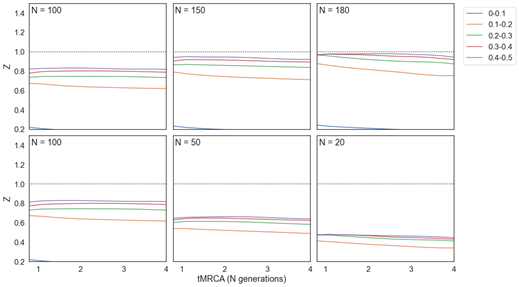

Supplement: S6 Fig — Each column is a separate set of simulations, with the top row plotting Z against tMRCA (measured in N generations, where N is the population size) for the larger daughter population, and the bottom row the smaller. There is no balancing selection and deleterious mutations are drawn from a gamma DFE with parameters inferred from D. melanogaster population data. Code to run these simulations can be found at https://github.com/vivaksoni/test_for_balancing_selection. DFE, distributions of fitness effect; tMRCA, time to the most recent common ancestor. (TIF) [file pbio.3001645.s009.tif]

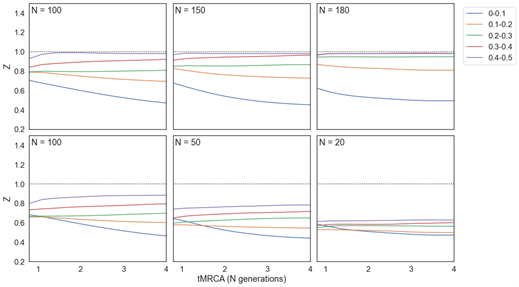

Supplement: S7 Fig — Each column is a separate set of simulations, with the top row plotting Z against tMRCA (measured in N generations, where N is the population size) for the ancestral population, and the bottom row the daughter population. There is no balancing selection and deleterious mutations are drawn from a gamma DFE with parameters inferred from D. melanogaster population data. Code to run these simulations can be found at https://github.com/vivaksoni/test_for_balancing_selection. DFE, distributions of fitness effect; tMRCA, time to the most recent common ancestor. (TIF) [file pbio.3001645.s010.tif]

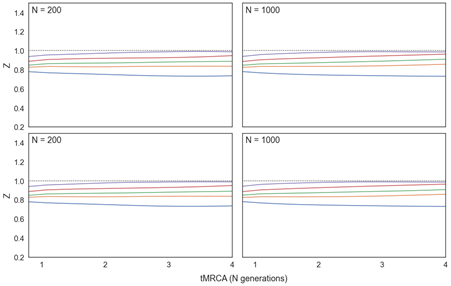

Supplement: S8 Fig — The ancestral population (of size N = 200) splits to form 2 daughter populations of size N = 100. Both daughter populations go on to expand in size. In the left column, the daughter populations double in size. In the right panel, they reach 10× their initial size. There is no balancing selection and deleterious mutations are drawn from a gamma DFE with parameters inferred from D. melanogaster population data. Code to run these simulations can be found at https://github.com/vivaksoni/test_for_balancing_selection. DFE, distributions of fitness effect; tMRCA, time to the most recent common ancestor. (TIF) [file pbio.3001645.s011.tif]

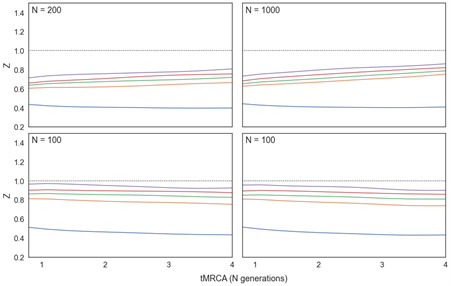

Supplement: S9 Fig — The ancestral population (of size N = 200) splits to form 2 daughter populations of size N = 100. One daughter population (upper panels) goes on to expand in size. In the left column, the daughter populations double in size. In the right panel, they reach 10× their initial size. There is no balancing selection and deleterious mutations are drawn from a gamma DFE with parameters inferred from D. melanogaster population data. Code to run these simulations can be found at https://github.com/vivaksoni/test_for_balancing_selection. DFE, distributions of fitness effect; tMRCA, time to the most recent common ancestor. (TIF) [file pbio.3001645.s012.tif]

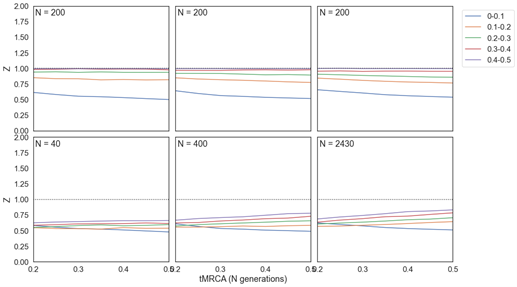

Supplement: S10 Fig — The ancestral population (of size N = 200) splits to form a daughter population of size N = 100, which expands to the final population size shown in the panel. Each column is a separate set of simulations, with the top row plotting Z against tMRCA (measured in N generations, where N is the population size) for the ancestral population, and the bottom row the daughter population. There is no balancing selection and deleterious mutations are drawn from a gamma DFE with parameters inferred from D. melanogaster population data. Code to run these simulations can be found at https://github.com/vivaksoni/test_for_balancing_selection. DFE, distributions of fitness effect; tMRCA, time to the most recent common ancestor. (TIF) [file pbio.3001645.s013.tif]

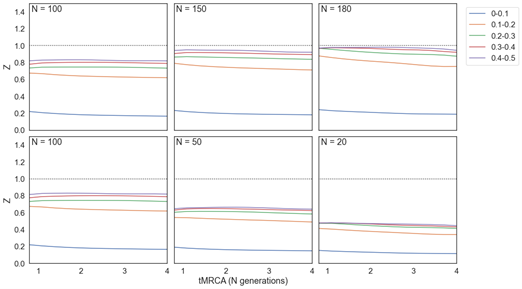

Supplement: S11 Fig — Each column is a separate set of simulations, with the top row plotting Z against tMRCA (measured in N generations, where N is the population size) for the larger daughter population, and the bottom row the smaller. There is no balancing selection and deleterious mutations are drawn from a gamma DFE with parameters inferred from human population data. Migration rate is 0.01 N. Code to run these simulations can be found at https://github.com/vivaksoni/test_for_balancing_selection. DFE, distributions of fitness effect; tMRCA, time to the most recent common ancestor. (TIF) [file pbio.3001645.s014.tif]

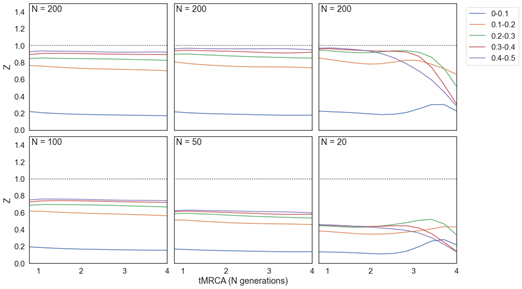

Supplement: S12 Fig — Each column is a separate set of simulations, with the top row plotting Z against tMRCA (measured in N generations, where N is the population size) for the ancestral population, and the bottom row the daughter population. There is no balancing selection and deleterious mutations are drawn from a gamma DFE with parameters inferred from human population data. Migration rate is 0.01 N. Code to run these simulations can be found at https://github.com/vivaksoni/test_for_balancing_selection. DFE, distributions of fitness effect; tMRCA, time to the most recent common ancestor. (TIF) [file pbio.3001645.s015.tif]

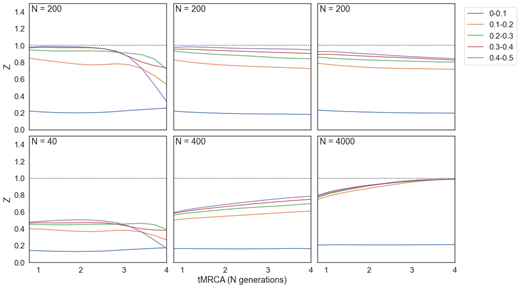

Supplement: S13 Fig — The ancestral population (of size N = 200) splits to form a daughter population of size N = 100, which expands to the final population size shown in the panel. Each column is a separate set of simulations, with the top row plotting Z against tMRCA (measured in N generations, where N is the population size) for the ancestral population, and the bottom row the daughter population. There is no balancing selection and deleterious mutations are drawn from a gamma DFE with parameters inferred from human population data. Migration rate is 0.01 N. Code to run these simulations can be found at https://github.com/vivaksoni/test_for_balancing_selection. DFE, distributions of fitness effect; tMRCA, time to the most recent common ancestor. (TIF) [file pbio.3001645.s016.tif]

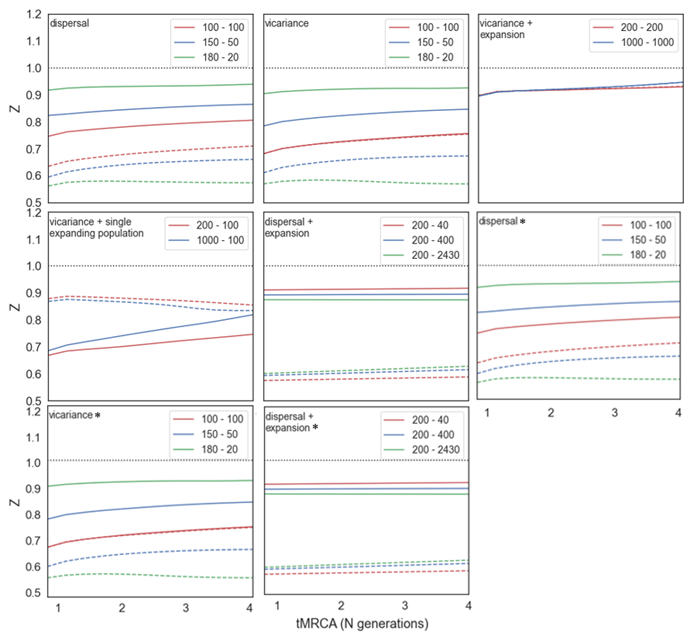

Supplement: S14 Fig — Each panel is a separate simulated scenario, with population sizes listed in the panel legend. (*) indicates simulations with migration (with migration rate 0.01 N). The first number is for the filled in data lines, denoting the ancestral population in dispersal scenarios, and for the larger population in the vicariance scenarios. The second number is for the dotted data lines, denoting the daughter population in dispersal scenarios, and the smaller population in the vicariance scenarios. For more details on each scenario, please see S1–S10 Figs. There is no balancing selection and deleterious mutations are drawn from a gamma DFE with parameters inferred from human population data. Code to run these simulations can be found at https://github.com/vivaksoni/test_for_balancing_selection. DFE, distributions of fitness effect; tMRCA, time to the most recent common ancestor. (TIF) [file pbio.3001645.s017.tif]

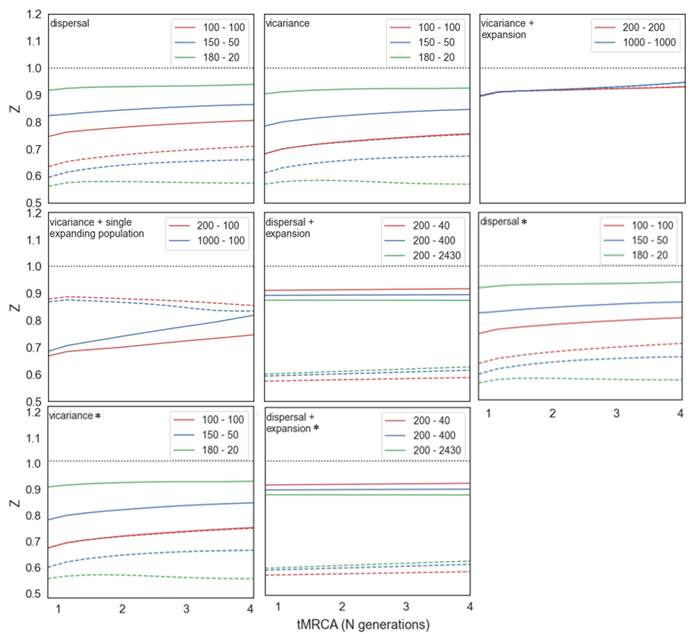

Supplement: S15 Fig — Each panel is a separate simulated scenario, with population sizes listed in the panel legend. (*) indicates simulations with migration (with migration rate 0.01 N). The first number is for the filled in data lines, denoting the ancestral population in dispersal scenarios, and for the larger population in the vicariance scenarios. The second number is for the dotted data lines, denoting the daughter population in dispersal scenarios, and the smaller population in the vicariance scenarios. For more details on each scenario, please see Supporting information S1–S10 Figs. There is no balancing selection and deleterious mutations are drawn from a gamma DFE with parameters inferred from D. melanogaster population data. Code to run these simulations can be found at https://github.com/vivaksoni/test_for_balancing_selection. DFE, distributions of fitness effect; tMRCA, time to the most recent common ancestor. (TIF) [file pbio.3001645.s018.tif]

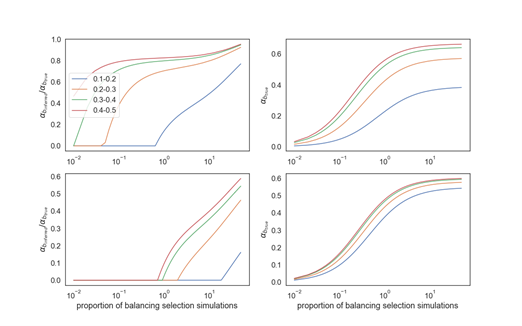

Supplement: S16 Fig — The top row plots are for the ancestral population, the bottom row for the daughter population. The left column plots αb inferred/αb true against the proportion of balancing selection simulations. The right column plots αb true against the proportion of balancing selection simulations. The 0–0.1 MAF category has been removed, and negative values have been truncated to 0 for the sake of clarity. Deleterious mutations are drawn from a gamma DFE with parameters inferred from human population data. Code to run these simulations can be found at https://github.com/vivaksoni/test_for_balancing_selection. DFE, distributions of fitness effect; MAF, minor allele frequency. (TIF) [file pbio.3001645.s019.tif]

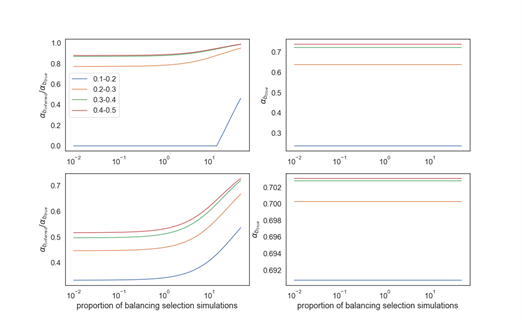

Supplement: S17 Fig — The top row plots are for the ancestral population, the bottom row for the daughter population. The left column plots αb inferred/αb true against the proportion of balancing selection simulations. The right column plots αb true against the proportion of balancing selection simulations. The 0–0.1 MAF category has been removed, and negative values have been truncated to 0 for the sake of clarity. Deleterious mutations are drawn from a gamma DFE with parameters inferred from human population data. Code to run these simulations can be found at https://github.com/vivaksoni/test_for_balancing_selection. DFE, distributions of fitness effect; MAF, minor allele frequency. (TIF) [file pbio.3001645.s020.tif]

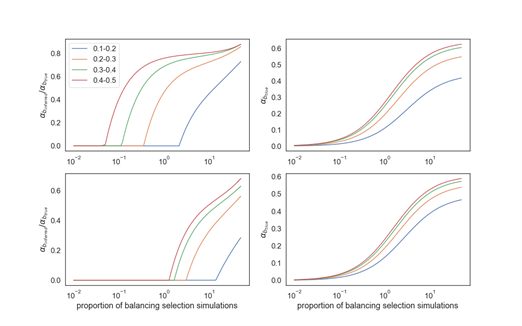

Supplement: S18 Fig — The top row plots are for the ancestral population, the bottom row for the daughter population. The left column plots αb inferred/αb true against the proportion of balancing selection simulations. The right column plots αb true against the proportion of balancing selection simulations. The 0–0.1 MAF category has been removed, and negative values have been truncated to 0 for the sake of clarity. Deleterious mutations are drawn from a gamma DFE with parameters inferred from human population data. Code to run these simulations can be found at https://github.com/vivaksoni/test_for_balancing_selection. DFE, distributions of fitness effect; MAF, minor allele frequency. (TIF) [file pbio.3001645.s021.tif]

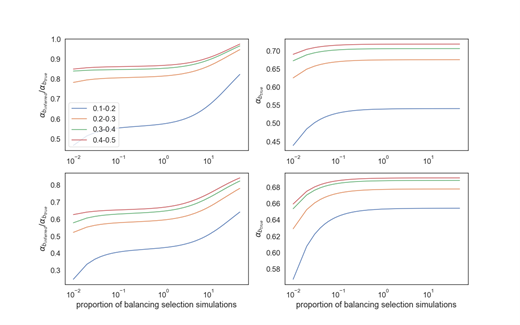

Supplement: S19 Fig — The top row plots are for the ancestral population, the bottom row for the daughter population. The left column plots αb inferred/αb true against the proportion of balancing selection simulations. The right column plots αb true against the proportion of balancing selection simulations. The 0–0.1 MAF category has been removed, and negative values have been truncated to 0 for the sake of clarity. Deleterious mutations are drawn from a gamma DFE with parameters inferred from human population data. Code to run these simulations can be found at https://github.com/vivaksoni/test_for_balancing_selection. DFE, distributions of fitness effect; MAF, minor allele frequency. (TIF) [file pbio.3001645.s022.tif]

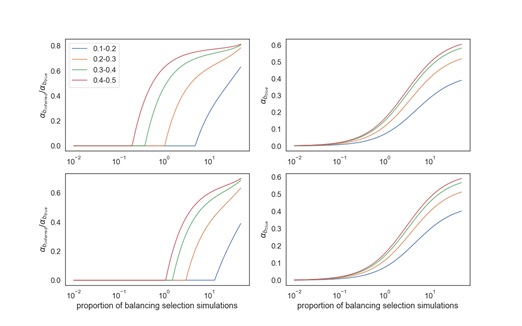

Supplement: S20 Fig — The top row plots are for the ancestral population, the bottom row for the daughter population. The left column plots αb inferred/αb true against the proportion of balancing selection simulations. The right column plots αb true against the proportion of balancing selection simulations. The 0–0.1 MAF category has been removed, and negative values have been truncated to 0 for the sake of clarity. Deleterious mutations are drawn from a gamma DFE with parameters inferred from human population data. Code to run these simulations can be found at https://github.com/vivaksoni/test_for_balancing_selection. DFE, distributions of fitness effect; MAF, minor allele frequency. (TIF) [file pbio.3001645.s023.tif]

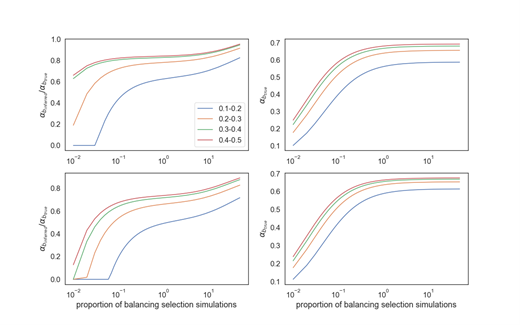

Supplement: S21 Fig — The top row plots are for the ancestral population, the bottom row for the daughter population. The left column plots αb inferred/αb true against the proportion of balancing selection simulations. The right column plots αb true against the proportion of balancing selection simulations. The 0–0.1 MAF category has been removed, and negative values have been truncated to 0 for the sake of clarity. Deleterious mutations are drawn from a gamma DFE with parameters inferred from human population data. Code to run these simulations can be found at https://github.com/vivaksoni/test_for_balancing_selection. DFE, distributions of fitness effect; MAF, minor allele frequency. (TIF) [file pbio.3001645.s024.tif]

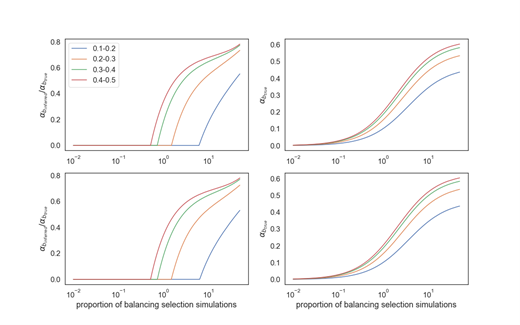

Supplement: S22 Fig — The top row plots are for 1 daughter population, the bottom row for the other. The left column plots αb inferred/αb true against the proportion of balancing selection simulations. The right column plots αb true against the proportion of balancing selection simulations. The 0–0.1 MAF category has been removed, and negative values have been truncated to 0 for the sake of clarity. Deleterious mutations are drawn from a gamma DFE with parameters inferred from human population data. Code to run these simulations can be found at https://github.com/vivaksoni/test_for_balancing_selection. DFE, distributions of fitness effect; MAF, minor allele frequency. (TIF) [file pbio.3001645.s025.tif]

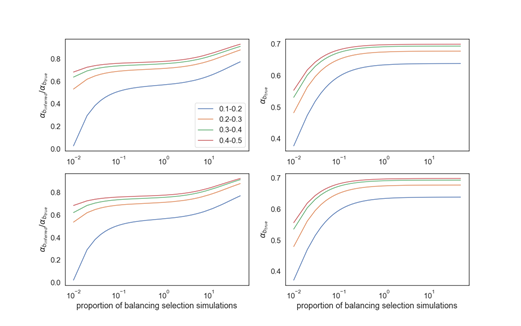

Supplement: S23 Fig — The top row plots are for 1 daughter population, the bottom row for the other. The left column plots αb inferred/αb true against the proportion of balancing selection simulations. The right column plots αb true against the proportion of balancing selection simulations. The 0–0.1 MAF category has been removed, and negative values have been truncated to 0 for the sake of clarity. Deleterious mutations are drawn from a gamma DFE with parameters inferred from human population data. Code to run these simulations can be found at https://github.com/vivaksoni/test_for_balancing_selection. DFE, distributions of fitness effect; MAF, minor allele frequency. (TIF) [file pbio.3001645.s026.tif]

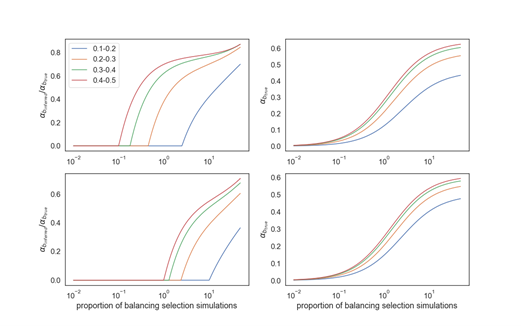

Supplement: S24 Fig — The left column plots αb inferred/αb true against the proportion of balancing selection simulations. The right column plots αb true against the proportion of balancing selection simulations. The 0–0.1 MAF category has been removed, and negative values have been truncated to 0 for the sake of clarity. Deleterious mutations are drawn from a gamma DFE with parameters inferred from human population data. Code to run these simulations can be found at https://github.com/vivaksoni/test_for_balancing_selection. DFE, distributions of fitness effect; MAF, minor allele frequency. (TIF) [file pbio.3001645.s027.tif]

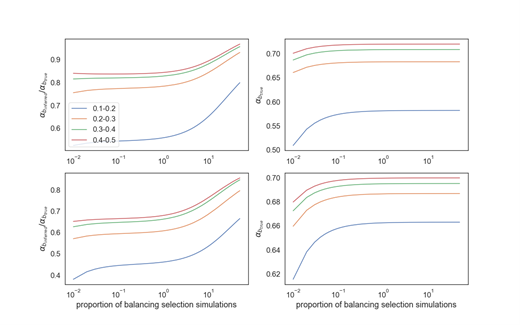

Supplement: S25 Fig — The left column plots αb inferred/αb true against the proportion of balancing selection simulations. The right column plots αb true against the proportion of balancing selection simulations. The 0–0.1 MAF category has been removed, and negative values have been truncated to 0 for the sake of clarity. Deleterious mutations are drawn from a gamma DFE with parameters inferred from human population data. Code to run these simulations can be found at https://github.com/vivaksoni/test_for_balancing_selection. DFE, distributions of fitness effect; MAF, minor allele frequency. (TIF) [file pbio.3001645.s028.tif]

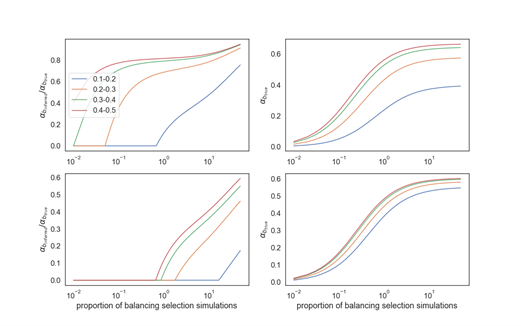

Supplement: S26 Fig — The left column plots αb inferred/αb true against the proportion of balancing selection simulations. The right column plots αb true against the proportion of balancing selection simulations. The 0–0.1 MAF category has been removed, and negative values have been truncated to 0 for the sake of clarity. Deleterious mutations are drawn from a gamma DFE with parameters inferred from human population data. Code to run these simulations can be found at https://github.com/vivaksoni/test_for_balancing_selection. DFE, distributions of fitness effect; MAF, minor allele frequency. (TIF) [file pbio.3001645.s029.tif]

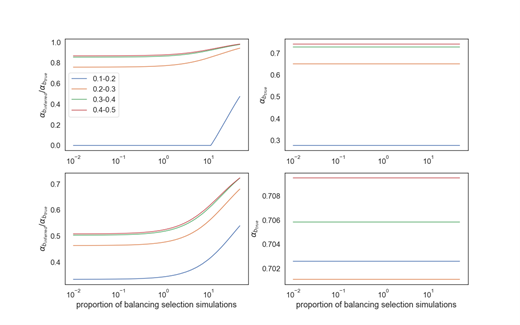

Supplement: S27 Fig — The left column plots αb inferred/αb true against the proportion of balancing selection simulations. The right column plots αb true against the proportion of balancing selection simulations. The 0–0.1 MAF category has been removed, and negative values have been truncated to 0 for the sake of clarity. Deleterious mutations are drawn from a gamma DFE with parameters inferred from human population data. Code to run these simulations can be found at https://github.com/vivaksoni/test_for_balancing_selection. DFE, distributions of fitness effect; MAF, minor allele frequency. (TIF) [file pbio.3001645.s030.tif]

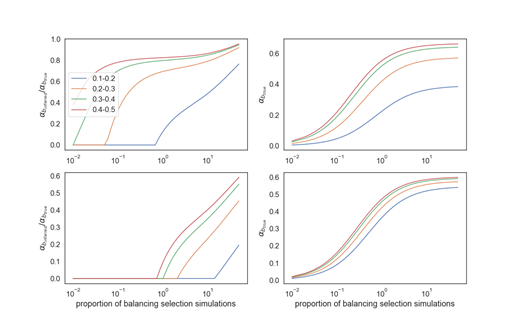

Supplement: S28 Fig — The top row plots are for the ancestral population, the bottom row for the daughter population. The left column plots αb inferred/αb true against the proportion of balancing selection simulations. The right column plots αb true against the proportion of balancing selection simulations. The 0–0.1 MAF category has been removed, and negative values have been truncated to 0 for the sake of clarity. Deleterious mutations are drawn from a gamma DFE with parameters inferred from human population data. Code to run these simulations can be found at https://github.com/vivaksoni/test_for_balancing_selection. DFE, distributions of fitness effect; MAF, minor allele frequency. (TIF) [file pbio.3001645.s031.tif]

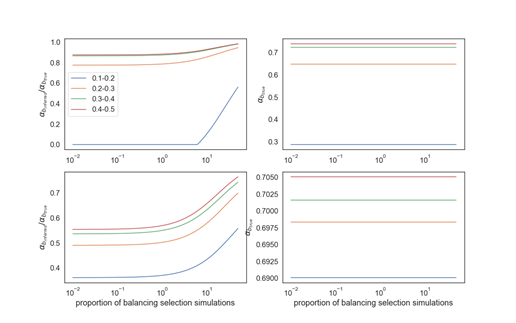

Supplement: S29 Fig — The top row plots are for the ancestral population, the bottom row for the daughter population. The left column plots αb inferred/αb true against the proportion of balancing selection simulations. The right column plots αb true against the proportion of balancing selection simulations. The 0–0.1 MAF category has been removed, and negative values have been truncated to 0 for the sake of clarity. Deleterious mutations are drawn from a gamma DFE with parameters inferred from human population data. Code to run these simulations can be found at https://github.com/vivaksoni/test_for_balancing_selection. DFE, distributions of fitness effect; MAF, minor allele frequency. (TIF) [file pbio.3001645.s032.tif]

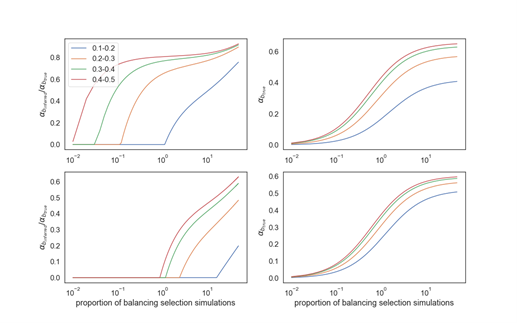

Supplement: S30 Fig — The top row plots are for the ancestral population, the bottom row for the daughter population. The left column plots αb inferred/αb true against the proportion of balancing selection simulations. The right column plots αb true against the proportion of balancing selection simulations. The 0–0.1 MAF category has been removed, and negative values have been truncated to 0 for the sake of clarity. Deleterious mutations are drawn from a gamma DFE with parameters inferred from human population data. Code to run these simulations can be found at https://github.com/vivaksoni/test_for_balancing_selection. DFE, distributions of fitness effect; MAF, minor allele frequency. (TIF) [file pbio.3001645.s033.tif]

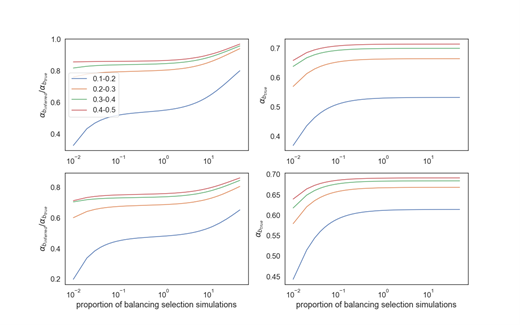

Supplement: S31 Fig — The top row plots are for the ancestral population, the bottom row for the daughter population. The left column plots αb inferred/αb true against the proportion of balancing selection simulations. The right column plots αb true against the proportion of balancing selection simulations. The 0–0.1 MAF category has been removed, and negative values have been truncated to 0 for the sake of clarity. Deleterious mutations are drawn from a gamma DFE with parameters inferred from human population data. Code to run these simulations can be found at https://github.com/vivaksoni/test_for_balancing_selection. DFE, distributions of fitness effect; MAF, minor allele frequency. (TIF) [file pbio.3001645.s034.tif]

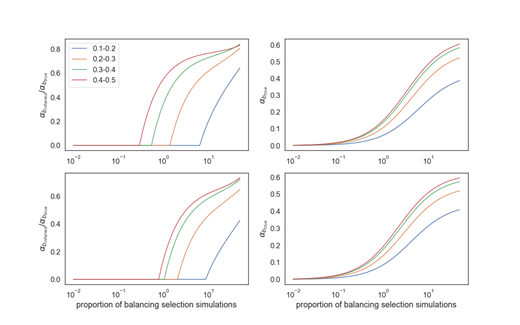

Supplement: S32 Fig — The top row plots are for the ancestral population, the bottom row for the daughter population. The left column plots αb inferred/αb true against the proportion of balancing selection simulations. The right column plots αb true against the proportion of balancing selection simulations. The 0–0.1 MAF category has been removed, and negative values have been truncated to 0 for the sake of clarity. Deleterious mutations are drawn from a gamma DFE with parameters inferred from human population data. Code to run these simulations can be found at https://github.com/vivaksoni/test_for_balancing_selection. DFE, distributions of fitness effect; MAF, minor allele frequency. (TIF) [file pbio.3001645.s035.tif]

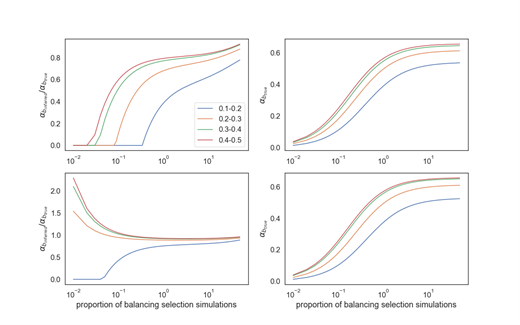

Supplement: S33 Fig — The top row plots are for the ancestral population, the bottom row for the daughter population. The left column plots αb inferred/αb true against the proportion of balancing selection simulations. The right column plots αb true against the proportion of balancing selection simulations. The 0–0.1 MAF category has been removed, and negative values have been truncated to 0 for the sake of clarity. Deleterious mutations are drawn from a gamma DFE with parameters inferred from human population data. Code to run these simulations can be found at https://github.com/vivaksoni/test_for_balancing_selection. DFE, distributions of fitness effect; MAF, minor allele frequency. (TIF) [file pbio.3001645.s036.tif]

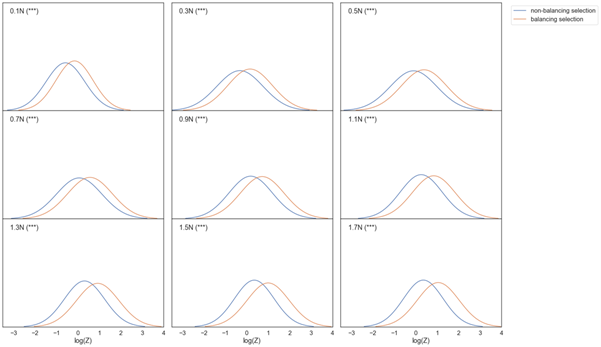

Supplement: S34 Fig — For each scenario 500,000 simulations were run. (*** p < 0.001 for a test between 2 distributions). Code to run these simulations can be found at https://github.com/vivaksoni/test_for_balancing_selection. (TIF) [file pbio.3001645.s037.tif]

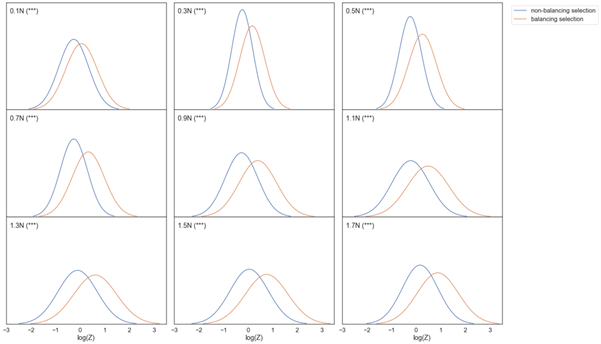

Supplement: S35 Fig — For each scenario 500,000 simulations were run. (*** p < 0.001 for a test between 2 distributions). Code to run these simulations can be found at https://github.com/vivaksoni/test_for_balancing_selection. (TIF) [file pbio.3001645.s038.tif]

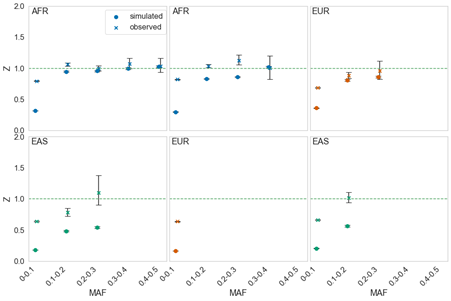

Supplement: S36 Fig — Shown are the observed (filled circles) and simulated (crosses) values of Z. Each column represents a different population comparison. From left to right: AFR and EAS, AFR and EUR, EUR and EAS. The population name in the upper left indicates which set of private polymorphisms are used to calculate Z in each population comparison. The x-axis represents private polymorphism minor allele frequency bins. Confidence intervals generated by bootstrapping. Code to extract and analyse the data can be found at https://github.com/vivaksoni/test_for_balancing_selection. The data underlying this figure can be found in S3 Data. AFR, Africans; EAS, East Asians; EUR, Europeans. (TIF) [file pbio.3001645.s039.tif]

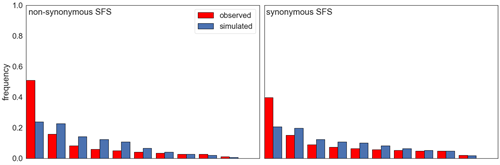

Supplement: S37 Fig — The SFS is summarised by combining SNPs at counts of 2 and 3, 4 to 7, 8 to 15…etc. with singletons considered by themselves. Code to extract and analyse the data can be found at https://github.com/vivaksoni/test_for_balancing_selection. The data underlying this figure can be found in S3 Data. (TIF) [file pbio.3001645.s040.tif]

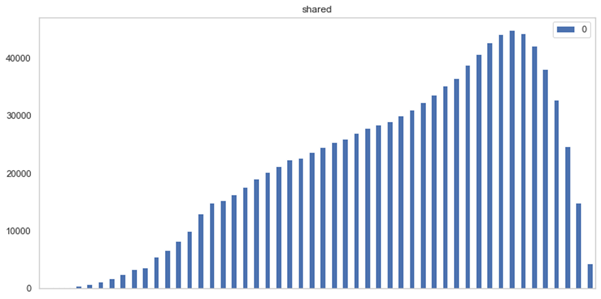

Supplement: S38 Fig — However, rare polymorphisms are more likely to be lost; the figure shows the average minor allele frequency of shared balanced polymorphisms in a simulation in which the population was duplicated and sampled N generations after the duplication event. Code to run these simulations can be found at https://github.com/vivaksoni/test_for_balancing_selection. The data underlying this figure can be found in S3 Data. (TIF) [file pbio.3001645.s041.tif]
