# Supplementary material for: A new test suggests hundreds of amino acid polymorphisms in humans are subject to balancing selection
Source: PLoS Biol. 2022 Jun 2;20(6):e3001645. doi: 10.1371/journal.pbio.3001645 (PMC9162324; doi:10.1371/journal.pbio.3001645)
Supplement: Attachment — Submitted filename: Replies to referee comments.docx [file pbio.3001645.s042.docx]

REVIEWERS' COMMENTS:

**Reply: we thank all referees for their constructive comments. One of the referees suggested investigating other methods of estimating the frequency of the shared polymorphism; we had previously used the mean frequency across the two populations, but simulations suggest using the frequency of the polymorphism in the population from which the private polymorphism is derived is less biased. We have therefore rerun all the simulations and data analysis using this method.**

Reviewer #1:

In this manuscript, the authors present a new method for detecting balancing selection and estimating the fraction of polymorphisms maintained by balancing selection. The method inspired by the McDonald-Kreitman test derives statistic Z, which compares shared and private polymorphisms between two populations to estimate an excess fraction of shared alleles that are maintained by selection. The test faces some interpretational challenges, similar to the MK test, such as the influence of deleterious mutations, especially on the low-frequency private variants, but also the impact of population-specific balancing selection on the statistic. These aspects are investigated either with simulations, or they are thoroughly discussed. I think the biggest weakness is uncertainty about the actual number of variants under selection, given that this number can vary a lot depending on the population size and number of deleterious alleles. Nevertheless, the analyses suggest that if
anything, the confounding factors underestimate balancing selection, but do not generate false positives. I feel that this method brings an interesting addition to the existing methods and adds new evidence to the view that balancing selection can be quite prevalent in the genome. 

Here I have two minor general comments and a few small specific comments to the text.

Estimated numbers of variants segregating under balancing selection are large approximations, given that Z and alpha can vary considerably depending on the population size, likely because of the prevalence of deleterious variants. On top of that, it's not clear what is the effect of the strength of balancing selection and the number of balanced polymorphisms on the estimate of alpha and the estimated number of variants under balancing selection. I was wondering how well the alpha estimates obtained in simulations relate to the true number of simulated balanced polymorphisms (=1) and how precisely can this method reflect the number of balanced polymorphisms in the populations.

**Reply: we have now added a section in which we consider how well alphaB estimates the true proportion of shared polymorphisms that are balanced. As expected alphaB underestimates the true value because of slightly deleterious mutations, and that this underestimation is greatest for the low frequency polymorphisms and when balanced polymorphisms are rare. However, the method performs fairly well under many other circumstances. This is now covered at the end of the section “Estimating the level of balancing selection”.**

Authors performed simulations with migration but they are not commented on in the manuscript and the results are not shown.

**Reply – we apologise for omitting these. They are now included.**

Line 174: In the legend description of subplots is unclear, I would suggest skipping "a) - orange" and "a) - green".

**Reply: this has been rewritten**

line 347: Please include the number of HLA and non-HLA genes that were used in the analysis in the text or the table legend.

**Reply: this has been added to the figure legend.**

line 350: The referenced figure (Fig. S13) is not the right one.

**Reply: corrected**

line 384: I think it should be: at least "one" population comparison.

**Reply: corrected**

line 384: Could you explain which populations are being compared here?

**Reply: we have added “comparing all pairs of populations”**

line 496: I think it should be: model "of" balancing selection.

**Reply: corrected**

line 513: I believe the reference should be figure S13 and S14 instead of S14 and S15.

**Reply corrected**

line 633: Please include the version of the program.

**Reply: now included**

line 637: Semicolon in superscript after the first citation.

**Reply corrected**

Supplementary Tables 2 and 3 are not referenced in the text, and Supplementary Table S1 is redundant with Table 3.

**Reply: all supplementary tables have now been removed since they are all redundant.**

To meet the requirements of the journal, please submit data or code to replicate the study.

**Reply: we have now done this, and these can be found at** [**https://github.com/vivaksoni/test_for_balancing_selection**](https://github.com/vivaksoni/test_for_balancing_selection)


Reviewer #2:

The MacDonald-Kreitman (MK) test has played a major role in molecular evolution studies. In the present paper the authors proposed a test similar in spirit to the MK test for balancing selection. Like the MK test it requires two types of sites that can be classified in two different ways. As in the MK test the two types of sites are generally synonymous and non synonymous changes. One assumes that the former evolve neutrally and the latter are under selection. The novelty in the present paper is to then consider polymorphisms that are shared between populations and polymorphisms that are private to one of the populations. So for the first class we will have S_S and S_N for synonymous and nonsynonymous changes at shared polymorphisms, respectively, and at the second one, we will have R_S and R_N. The authors then reasoned that under balancing selection one will have:

Z=S_N/S_S/R_N/R_S >1

They then went on to show that an estimate of the proportion of non-synonymous mutations subject directly to balancing selection is

alpha_b=1-S_S*R_N/S_N*R_S

There is no question that this is a neat and clever idea and I enjoyed reading the paper. Most of the paper is devoted to simulations to test factors influencing Z and alpha_b and to an application to human data. My main concerns are (i) that the simulations are presented in a way favourable to the test even though there is generally a caveat at the end of each paragraph stressing the limitations and (ii) the lack of an explicit statistical framework. I would have liked the paper to be divided in three parts: (i) simulations to investigate the behavior of Z and alpha_b, (ii) development of a statistical test at the genome level and at individual genes level, (iii) application to a human data set. The second part is simply completely lacking. Also, if possible it could be good to apply the method to a couple of other species with different life history traits and demographies. It could also be good to clarify what the Z statistics or alpha_b mean at the genome level vs individual gene level. What does it mean that evidence of balancing selection is observed globally but not at single loci? Is it simply a statistical power issue or could it mean something else?

**Reply: we have now included a new section, “Statistical Tests” in which we address how the test should be performed. At the single gene level, a simple 2x2 chi-square heterogeneity test is warranted since synonymous and non-synonymous sites share the same genealogies. For, tests involving multiple genes we suggest performing the test by non-parametric bootstrapping, bootstrapping at a level that includes all sources of variance. Unfortunately, we haven’t had time to apply the test to other species. The difference between the genome and gene level analyses is definitely a problem of power. This test is very poorly powered to do anything at the single gene level.**

Minor comments:

lines 209-210: why not call Watterson coefficient a_n as usual or give it explicitly?

**Reply: we have now corrected this.**

line 258-263: the statement feels a bit contrived.

**Reply: we have rewritten this section; we just want to make the point that not observing Z>1 in both comparisons is not evidence that there isn’t balancing selection.**

Line 496-97 A more realistic model OF balancing selection

**Reply: corrected**


Reviewer #3:

The manuscript of Soni et al describes a statistic to measure balancing selection in recently diverged populations inspired by the McDonald-Kreitman test. With some assumption, they present a derivation that uses this statistic to estimate the total number of alleles that are subject to balancing selection between two populations. Simulated data from two-population models were used to evaluate the measurement under several two-population scenarios. Subsequently, the statistic was applied to human genomics data using pairs of 1000 Genomes continental population groupings. The authors report evidence of balancing selection in these populations, particularly in continental African population groups relative to others. Finally, using GO-enrichment categories, gene groups were analyzed separately to identify specific types of genes with evidence of balancing selection. Where sample size allowed, these analyses were further narrowed down to identify individual genes with variants
under balancing selection.

This manuscript proposes a statistic with two novel implications: (i) a measure of balancing selection better suited to detection on smaller time scales (thousands of years, rather than millions of years), and (ii) a direct estimate of the number of loci maintained by balancing selection in a given region between two populations. Either improvement could easily complement existing methods and approaches that are available (and the authors cite). However, with the analyses presented, the authors do not provide sufficient evidence supporting either of these two inferences. Specifically, the absence of power analyses made the utility of the Z statistic hard to interpret. Furthermore, the performance of the method was difficult to assess without positive controls or comparisons to other existing techniques. Concerns are described in the comments that follow.

\## Major Concerns:

1. A proposed framework for the test(s) and statistical inference of the quantities of interest. The authors lay out a statistic and some motivation, and then how it could be used to infer specific quantities of interest, but the presentation was hard to grasp as the presentation was relatively informal. We would strongly encourage the authors to organize and present a formalized approach - inference of the statistic, the test in question, the null hypothesis (and interpretation), then a proposed test, how significance of the test will be assessed.

**Reply: we have now added a section on statistical tests, which addresses the referee’s concerns about how the test should be applied. Otherwise we feel that the presentation of the test is logical; we start by presenting the basic idea behind the test in the introduction; we then demonstrate by simulation that the test has the potential to reveal balancing selection before analysing the extent to which it is robust to departures from the assumptions of the model. We now include a section on how the test should be applied.**

1a. For example, the values of Z described in various figures in the manuscript are not easily interpretable -- what does the magnitude of Z mean?

**Reply: we agree that Z does not have a clear meaning and we have considered rephrasing the manuscript in terms of alphaB. However, we think it is better to retain Z as the test statistic, and alphaB as the interpretation of this test statistic under a particular evolutionary model; this obviates the difficulty of interpreting negative values of alphaB, which have no logical meaning.**

1b. What is the proposed test, and the null hypothesis? It seems the authors would mean Z > 1 is the alternative; so does that imply that Z < 1 is the null? What is the actual test used to determine that this is the case?

**Reply: indeed Z<=1 is the null. We have now clarified this and how to apply the test in the section “statistical tests”**

1c. In the latter half of the paper, the authors calculated bootstrapped confidence intervals which may be the manner in which they would like to determine significance, but the procedure for these calculations were not well described. Again, it seems important to organize all of this into a single place with sufficient methodological details that the approach can be evaluated clearly (and is clear to the reader).

**Reply: how we derive the confidence intervals is now made clear in both the main text and the Material and Methods; our justification for bootstrapping by gene is given in the section “statistical tests”.**

1d. The simulation results were particularly glaring in this regard. It was not clear as to how this was done (i.e., everything should be in methods, organized succinctly with reasoning, rather than in legends). It seems important that there needs to be some sort of confidence interval estimate of variance for Z to assess if Z is meaningfully different from 1, and how this could be shown.

**Reply: we don’t present confidence intervals, but have run the simulation sufficiently often to reduce the standard error of our estimates to very low levels.**

2. A formal, statistical workup of the proposed test(s) and inference. After the authors lay out a specific inference procedure, it seems incumbent then to more deeply explore the statistical characteristics of this statistic and their test. Without this, it is difficult to assess what the calculated scores actually quantify and to understand how well the proposed method works in practice.

2a. Power analyses. Is the method able to accurately identify balancing selection? Without a deeper interrogation of the power of the statistic, it is difficult to determine the utility of the method. While it is claimed that the method is robust with respect to generating false positives, no assessment is made to what extent the method is powerful enough to determine true positives. Parameters that might influence power could include (but are not limited to): survey sample sizes; TMRCAs, demographic effects; recombination and mutation rate variation; when a balanced allele arises and selection parameters that relate to its equilibrium frequency, different balancing selection models, etc.

**Reply: although an interesting area we have not pursued the question of statistical power. As the referee suggests there are so many factors that might influence power. To even scratch the surface here would take a huge amount of work and would ultimately yield little insight; there are clearly going to be situations where the method has reasonable power, and other instances where it has little power.**

2b. Error rate evaluation. While the authors present simulation and show the trends of various Z statistics under various demographic two-population scenarios, this feels like only a start to assess how often under various circumstances one might observe Z > 1 without balancing selection. Right now, the manuscript does not formally assess the error rate of the proposed approach (because there is no proposed test formally, and how often type 1 errors are made).

**Reply: we have evaluated a range of simple demographic models and find that Z is significantly less than 1 when there is no balancing selection. The universe of demographic models is however infinite so it’s unclear how to progress beyond this.**

2c. Properties of inference of number of balanced alleles. In particular, a statistical workup under the alternative for inferring the number of balanced alleles was notably absent in this presentation. They present estimates (with confidence) on real data, but without any workup where the truth is known, it is impossible to know how accurate/biased these estimates are in practice (and the authors admit for various reasons this approach may have bias). Simulations that answer this question would have significantly strengthened the claims that this method produces accurate estimates for both Z and the number of balanced polymorphisms. How far off is the method's estimate from the "true" number of balanced polymorphisms? Does the procedure to generate confidence intervals capture the truth at the expected level (90% CIs capture the true value 90% of the time)? Does this depend on the level of recombination or local LD (as the author's claim is a limitation in the
introduction)?

**Reply: we have now performed simulations with and without balancing selection and considered the estimation of alphaB. In most circumstances alphaB is substantially underestimated. This is now included at the end of the section on estimating alphaB.**

2d. No comparison to previous methods. While the proposed method is claimed to excel where current methods struggle (robust to shorter divergence times), this presentation makes no attempt to compare to any existing methods and compare them to the proposed statistic despite the authors being aware of many methods (they cite many in the introduction). An evaluation on simulated data comparing performance would have strengthened the argument that this method is truly an improvement on currently existing methods, and thus a novel contribution to the field.

**Reply: this would have been an interesting exercise but unfortunately we have not had time to do this. We regard our method as an addition to the methods available; it clearly has some strengths – simple and yielding a direct estimate of the number of polymorphisms subject to balancing selection – but underpowered to say anything about individual genes.**

3. Novelty. It could be that there is methodological or biological novelty here. However, the current presentation makes what is, in fact, novel extremely challenging to ascertain. 

3a. Defining methodological novelty. Is the proposed method simply McDonald-Kreitman? If not, the authors should explain exactly how/why (if the MK test is motivating) and point out the precise differences or the intuitive way they got to the inference procedure they presented. Moreover, the estimate of the proportion of balanced sites could be novel; however, without a workup of its merit (read: statistical assessment of accuracy etc.), it is hard to know if/how meaningful this is.

**Reply: The McDonald-Kreitman test was originally proposed as a test of whether the divergence between species had been affected by adaptive evolution; our variant on this test is apply this to polymorphisms that are shared between species. It is noteworthy that several groups have considered shared polymorphisms as evidence of balancing selection, but none, have come up with this simple method to control for neutral polymorphisms that might be shared because of common ancestry. The method is novel, but it finds its inspiration in a very similar and commonly used test.**

3b. Defining biological novelty. There is quite a bit of literature now which reports statistics that have been applied to 'scan' the human genome for selection. What, if any, loci do the authors highlight that have not been reported? No new genes were considered significant after Bonferroni corrections. There were no substantial discussions of new genes that were implicated with this new method. This itself diminishes the potential interest of the current work. However, if there exists some angles of novelty, we encourage the authors to really bring that forward in presentation. E.g. Specific examples, regional plots, to guide the intuition about the signal that the author's method is sensitive to (and perhaps why their method reveals it whilst others do not).

**Reply: the method is not highly powered to say anything about individual genes, but it can yield a simple estimate of the number of polymorphisms that are subject to balancing selection. Understanding the overall prevalence of balancing selection is just as interesting, in our opinion, as knowing which genes are subject to the process. We do discuss the cases in which there does appear to be a signal of balancing selection and we compare our results to some previous analyses in the discussion.**

4. Details about how the method was ultimately applied were missing or hard to find. What values were used for set parameters such as the choice of Watterson's coefficient or the proportion of neutral non-synonymous mutations? How were the number of neutral mutations maintained in LD set? Furthermore, the details for the bootstrapping procedure were also unclear. In data analysis, were windows used to analyze the data, and if so, what was considered (and why; does this choice influence power?)?

**Reply: we provide the parameters in our simulation including locus size, population size, recombination and mutation rates. These ultimately determine the numbers of polymorphisms in LD. We have now clarified how the statistical tests were conducted including the bootstrapping procedure. The method does not use windows; we have now made this clear.**

\## Minor Comments:

5. The structure of the paper made it difficult to read. The logic of these types of papers approximately follow a theory, simulation/statistical workup, application to real data presentation flow. This manuscript has points that seem to jump across a lot of these spaces, making it confusing to read through.

**Reply: the structure of the manuscript is as the referee suggests; we introduce the test in the introduction; we construct a simple statistic that captures this signal and confirm by simulation that this signal is present when there is balancing selection. We investigate whether there are scenarios under which our test could give erroneous conclusions, and finally we apply the test to some real data**

6. Figures are difficult to interpret. Figure 1 was hard to parse and the figure caption confusing. The merging of descriptions for different panels was difficult to follow and the sharing of figure legends was not intuitive. Furthermore, the lack of variance on the simulation results, as described previously, detracted from the plotted point estimates. Several of the other figures were also a bit challenging to understand.

**Reply: we have now rewritten the figure legend. We have also made it clear that we ran sufficient simulations that there is almost no error in our estimates.**

7. Point of discussion, in the intro it is true that several methods are computationally intensive and do require simulations and/or knowledge of demography to maximize power. However, not all methods do that. I would suggest some caution about how the authors report this (L50 - 52) different investigators might differently disagree with how they characterize their methods! (which is why the author's point about estimating the number of sites could add value).

**Reply: thanks; we have now rewritten this sentence – we have simply stated that most methods require simulations to derive the null distribution.**


Reviewer #4:

"A new test demonstrates that balancing selection maintains hundreds of non-synonymous polymorphisms in the human genome", by Soni, Vos and Eyre-Walker is an interesting manuscript that addresses an interesting question: how many polymorphisms are maintained by balancing selection? As the authors note, how much variation is maintained by natural selection is a fundamental question in population genetics and evolutionary biology. The authors have modified a classical method to address this question. This is an excellent attempt at quantifying the effects of balancing selection. The authors then apply their method and conclude that balancing selection maintains more than a thousand non-synonymous polymorphisms in humans. This means that thousands of genes are under balancing selection in humans and that balancing selection is fairly common. This result is surprising as the current view in the field is that balancing selection is quite rare. If accurate the conclusions would be not only surprising, but also interesting and very important. They would have important implications and ramifications in our view of the evolution of polymorphism. I am very positive about this manuscript. Yet the authors do not show that they have explored the behaviour of the test in a way that establishes their estimates are clearly realistic. With a bit of extra work they can discard alternative possible explanations for their observations and make their results and their arguments convincing.

Major points:
- Introduction: The method is very different from existing methods in multiple ways. Z has many advantages over other methods, as stated in the manuscript. But also important disadvantages, the most important being:
1. That it is not adequate to identify individual genes, and 
2. That it cannot identify selection at non-coding loci. 
This should be more clearly stated, preferably in the introduction.

**Reply: we now make the points the referee suggests in the discussion.**

For 2. the authors should mention how much of an issue this is expected to be. Are most balanced polymorphisms non-synonymous, or regulatory? What did the best previous methods that are not limited to non-synonymous changes find in this regard?

**Reply: the method can potentially identify balancing selection in non-coding DNA if sites can be divided into those that are likely functional, and those that are not; this should be possible by dividing sites into TF and non-TF binding sites for example. Whether most balancing selection is in non-coding or protein coding sequences remains unknown I believe.**

- L 147: I understand the motivation to divide polymorphisms in bins of frequency. This has the advantage that the age of the alleles in the shared and private categories is similar, which I think addresses many potential issues. If the authors agree that this is an advantage, they could state this more clearly. But for shared polymorphisms the frequency used is the mean frequency in the two populations. This potentially moves the polymorphisms across bins of frequency compared with where they are in the population used for the private polymorphisms, in a way that is difficult for me to predict and that may affect K. Averaging could move non-synonymous polymorphisms to a higher frequency bin than they have in the population where private polymorphisms are counted, for example if purifying selection is less efficient in the other population. That would inflate Z in ways that seem consistent with patterns in Figure 2. But rather than me arguing about expectations, it is easy to explore how averaging frequency affects Z when populations have different Ne and whether this is an issue.

**Reply: the referee’s comment prompted us to reconsider how we calculate the frequency of the shared polymorphism. We had previously considered the average frequency across the two populations. Our simulations suggested that this did not yield artefactual evidence of balancing selection. However, we have investigated whether simply using the frequency of the shared polymorphism in the population from which the private polymorphisms are drawn might be better, and we find that this generally gives less biased estimates of the number of balanced polymorphisms. We have therefore rerun the simulations and data analysis using this alternative method. Unfortunately, our results become less significant as a result.**

- I believe that LD to a balanced polymorphism must increase global Z. Not in the simulations because exons are individually simulated. But in real data where a single balanced polymorphism can maintain multiple non-synonymous variants through linkage in different exons of the same gene. This could potentially explain the higher Z in regions of low RR. If this is the case, the observed Z could be substantially overestimated. One could discard this possibility by analysing only one exon per gene.

**Reply: actually, the opposite is true; Z is related to the proportion of shared polymorphisms that are being directly maintained by balancing selection, rather than being in LD. Non-synonymous variants in LD with a balanced polymorphism reduce Z so this is unlikely to explain the low RR results.**

- Figure 1: I cannot see that "Z increases as a function of the time since the population split". There is no change in many lines, and a modest one in others. Such a modest effect is surprising because as the authors mention one would expect to observe such an effect.

**Reply: we have edited this because as the referee suggests, there is no evidence for this, which is suprising.**

- Demography influences Z. This being the case, in my opinion more efforts are needed to show that demography cannot explain the results, but under two simple models and a human demography they do not observe Z>1. Two issues I see:
1. The best approach would be to ask which models could potentially inflate Z and test them. The authors will be better at this than me, but many population size changes over time such as expansions and bottlenecks seem to have that potential. Simulation results for those two, and other models that can potentially inflate Z, would convince the reader that results are not due to differences in demography among two populations.

**Reply: there are potentially demographic models that might cause an inflation of Z. We have explored one model that might cause this– one in which there is population size increase since the populations split. We find that this does not yield artefactual evidence of balancing selection. There may be other models such as the fluctuating population model.**

2. Even if demography could not by itself generate Z>1, by inflating Z it would affect the estimates of the number of non-synonymous polymorphisms maintained by balancing selection. Is this considered in the estimates presented?

**Reply: this is correct, but generally Z is underestimated. We have now included a section in which we mix simulations with and without balanced polymorphisms and compare alphaB to its true value.**

- L 238: The human populations analysed are presented in terms of continental groups, but there are multiple populations in each of these groups. Was one population chosen per group, or was the combination of all populations in each of these groups analysed? In the latter case combining differentiated populations affects allele frequencies and could influence Z. I presume that the authors have considered this but it would be good to address this point and add this information to the methods.

**Reply: we summed data from the sub-sub-populations to yield are four sub-populations. We have now made this clearer in the methods section.**

- Figure 2: Are there no values for Eur for the Africa/Europe comparison for 0.1-0.5 in Figure 2?

**Reply: the estimate of Z can be subject to a large sampling variance unless there are substantial numbers of polymorphisms, so we require there to be at least 20 of each type of polymorphism before we plot the value; this is now made clear in the figure legend.**

- Differences among populations and among analyses using a different population for the private polymorphisms are difficult to understand. I think that at least potential explanations should be provided. The case of EAS/SAS is odd. Z is positive on EAS but very negative in SAS. Are these two mirror images of the same pattern? Such pattern seems present in all plots and not expected from balancing selection as far as I can see. Could it be due to population differences in Ne? Or other technical effects? This is an important point needs to be addressed. I don't think that the discussion in L. 525 is convincing because it does not address this issue but points to a different argument, that in the limited set of simulated models Z was never >1.

**Reply: generally, we find that Z differs between the populations when we have two populations with different demographies; i.e. Z tends to be downwardly biased, but its biased to different extents depending on the demography.**

- The analysis per gene category is very interesting and potentially informative. Many highlighted categories seem to contain very long genes, which is worrying. It would be easy to test if these genes are longer than expected or use a method that controls for gene length, to ensure that technical artifacts are not influencing the result.

**Reply: this is an interesting observation which we have not had time to pursue.**

- The analysis per gene seems so underpowered to become almost uninformative. First, what is the behaviour of the test when polymorphisms are binned as in this analysis? Second, as there are no significant signatures genes are chosen by how many times they have Z>1, but as noted in the text these analyses are not independent. I don't see much value in the analysis of individual genes but if the authors decide to keep it should start with a strong statement about the limitations of the method for this application.

**Reply: we would agree that the method is under-powered, and as such we regard the single gene analyses as merely suggestive that there might be balancing selection.**

- Associated overdominance is an interesting possible mechanism, but it is unclear why it is singled out if the authors do not think it is likely. I can guess, but it is not clear from the text.

**Reply: we realised that associative overdominance could not explain our results, because it would tend to affect non-synonymous and synonymous mutations equally; i.e. associative overdominance arises when there are two deleterious recessive mutations segregating at two loci that are close to each other (e.g. a and b are recessive to A and B). If there are two neutral or selected alleles at a third locus (C and c) and each of these alleles is associated with one of the two recessive alleles (e.g. C tends to be in LD with a, and c with b), then the fittest genotype at the C locus is Cc. The alleles at this third locus might be synonymous or non-synonymous.**


Minor points:
- There are some track changes still in the text.

**Reply: corrected.**

- The paper is well written and generally clear, but not easily accessible to a general PLoS Biology audience. The authors could explain more clearly the effects of demography and selection on allele frequencies and the test for the non-expert.

**Reply: we are cautious about expanding the explanations given the length of the manuscript already.**

- More methods details need to be provided, especially in the Results. For example, in L122 the reader should be able to know what distribution of s is used, and more details on the demographic model used.

**Reply: more details have been provided.**

- I am a bit confused about the effect of linkage on the method. My understanding is that linked alleles are not counted in the non-synonymous polymorphisms claimed to be maintained by balancing selection in the genome. But there must be non-synonymous polymorphisms that are maintained due to linkage to the polymorphism under balancing selection. Can the authors estimate the total number?

**Reply: we have not pursued this due to time constraints.**

- Figures. I advice that titles are added to the figure, or at the very least the figure panels are numbered.

**Reply: titles have now been added.**

- The population size in the simulations is low. How strong was s so the balanced alleles were not lost due to drift?

**Reply: the strength of selection is now specified.**
