# Supplementary material for: A new test suggests hundreds of amino acid polymorphisms in humans are subject to balancing selection
Source: PLoS Biol. 2022 Jun 2;20(6):e3001645. doi: 10.1371/journal.pbio.3001645 (PMC9162324; doi:10.1371/journal.pbio.3001645)
Supplement: Attachment — Submitted filename: Replies to reviewers comments.docx [file pbio.3001645.s043.docx]

REVIEWERS' COMMENTS:


Reviewer #1:

I am satisfied with the authors' revision. I like the idea behind the method, and although the exact estimates of variants under balancing selection have to be taken with caution, I think the most critical issues of the method are thoroughly explored or discussed by the authors.

**Reply: we thank the referee.**


Reviewer #2:

This is a revised version of a paper that I reviewed earlier on. The new version has been improved and the authors have satisfactorily taken my comments into account. The "statistical test" part could have been a bit longer and it would have been nice to see the test applied to another species than humans but this does not diminish the novelty and the potential interest of the approach.

**Reply: we are sorry that it was not possible to apply the test to another species. It took us a long time to get to this point and we ran out of time.**

Minor comments:

Line 124: Yates et al, 2020 is missing from the Reference list

**Reply: corrected.**

Line 256: remove "is".

**Reply: corrected**


Reviewer #3:

The authors provide thoughtful responses to many of the previous comments and expand simulations in several places to provide additional clarity about the approach and its properties, in particular demographic simulations to describe the expected value of Z, and the (biased) properties of the inference of alpha (the proportion of sites expected to be subject to balancing selection). 

That said, some lingering concerns remain given the current presentation. I still think the most obviously important part of the paper (and most novel) is around the estimate of alpha, even though it carries with it substantial bias (underestimated). 

My concern focuses here on the utility and capabilities of Z as a gene-level test. 

In the response to my comments and in the manuscript, the authors state that the method for single gene analysis is underpowered. I point out that there is still not a formal quantification of this. 
In response to this comment in the previous review (comment #2a), the authors seem to agree that many factors would influence power, but that this would "take a huge amount of work" which would "yield little insight" even though they state (without evidence) that they believe there are certainly situations "where the method has reasonable power". It also remains unclear if this approach does better than any previously published method, which brings with it concerns (in general) about utility.

**Reply: the referee is correct, we were really being guided by intuition here. We have now added some simulations (see below).**

To try to be fair to the authors here (and trying to step past particularities of how unconvincing this response is in this case), I suggest two approaches that ought to be straight-forward and would assuage some of my concerns over this point. My hope is that these should not be so onerous on the authors that they could not be accomplished. These would also allow the authors to be fair about the potential of their method, and readers could evaluate what is shared and determine if it is useful for their purposes. (In response to Comment 2D, the authors seem to argue that their method is "an addition to the methods available… underpowered to say anything about individual genes" but main utility could be around direct estimates of the number of polymorphism subject to balancing selection).

(i). Extensive simulation work has been undertaken which is very helpful. However, it seems that these report the E[Z] along with the associated standard error of the mean, which is so small as to be negligible).
Since they have the variance (needed for the error calculation), the authors could - without much extra work - present the distribution of Z under some simple basic demographic null (e.g., those with -ve selection Fig 1b say, and/or a small subset of the most important from Supplementary figure S1-S13) and alternative +ve balanced selection (Fig 1a, say). Intuitively, the more that alternative Z distribution overlaps with a given distribution of Z under a given demographic/-ve selection scenario without selection indicates reduced power; less overlap suggests more power. Thus, some description of the variance of Z in those scenarios would give some intuition about power even if one did not formally make calculations.

**Reply: this is a nice suggestion and we have now done this. We have run simulations of a gene with average human dimensions into which we introduce a balanced polymorphism in each of 8 exons, under the simplest demographic model – the ancestral population size splits into two. We consider the distribution of Z values as the referee suggests. We also repeated the analysis for a gene that is ten-fold larger than the human average. In both cases the distribution of Z values overlaps quite considerably between those genes with balancing selection and those without, thus demonstrating that the test has little power at the single gene level.**

(ii). Many alternative methods exist. While perhaps a full characterization of power across all methods is certainly overkill (especially if this version of the method is not well powered), choosing a *single* one - an approach they could choose that minimizes difficult of use while maximizing prior power - with appropriate power analyses and assessing performance in comparison to the author's statistic would both add some quantification to the "underpowered" assertion, while also serving as a natural way to emphasize the novelty and impressive achievement of this statistic, namely the alpha estimate.

**Reply: all other methods focus on single loci, and many have been subject to independent power analyses by their original authors. Given the low power of our method we do not think it would add very much to compare methods; our method has almost no power, other methods do have power. Furthermore, selecting one is problematic. We now explain why we think our method will be underpowered relative to other methods for single genes – other methods leverage the effects that balancing selection has on linked genetic variation, whereas our method cannot use this information; in fact, it is confounded by it.**

\## additional minor comments
- L122 - what does "density of balanced polymorphism" mean? 

**Reply: we have now clarified this with the statement “i.e. the number per bp”**

- What is the expected stationary allele frequency for figure 1a for the balanced site(s) given the parameters? They should report (esp. since they partition by allele frequency later).

**Reply: in these simulations we generated balanced polymorphisms where the expected distribution follows a uniform distribution; however, because rare balanced polymorphisms are lost more readily, the realised frequency distribution is different to this. This is now made clear.**

- I appreciate the workup of S1-S13 presented. Given what they report, I have to admit that I am somewhat surprised that there does not appear to be a single scenario where the E[Z] is not greater than 1. In my experience, just about every form of selection has some confounding demographic scenario whereby a method that attempts to infer selection must try to reconcile. I scratched my head about this for a little while and couldn't immediately think of something. That said, I would strongly encourage the authors to think hard and out-of-box about scenarios that might actually results in Z > 1 in expectation.

**Reply: we agree that there is probably some demographic scenario that will generate false positives; we have simulated the most obvious, which is population size expansion, and its possible if you push the model really hard, it will fail. However, as we explain the test may be more robust because we are comparing two types of polymorphism.**

E.g., I feel like maybe recent introgression from a (say) hominid ancestor (something that data is increasingly indicating may be a possibility) might be one of those cases?

**Reply: we have simulated some scenarios involving migration and find no evidence that Z>1. This is probably because introgression and migration increase the frequency of both synonymous and non-synonymous shared polymorphisms.**

- L792-794; L934-940; L941-944: Check double reference in citations

**Reply: corrected.**

- L256: typo, extra 'is'

**Reply: corrected**
